# A system model of the effects of exercise on plasma Interleukin-6 dynamics in healthy individuals: Role of skeletal muscle and adipose tissue

**DOI:** 10.1371/journal.pone.0181224

**Published:** 2017-07-12

**Authors:** Micaela Morettini, Maria Concetta Palumbo, Massimo Sacchetti, Filippo Castiglione, Claudia Mazzà

**Affiliations:** 1 Interuniversity Centre of Bioengineering of the Human Neuromusculoskeletal System, University of Rome “Foro Italico”, Rome, Italy; 2 Institute for Applied Calculus “Mauro Picone”, National Research Council of Italy, Rome, Italy; 3 Department of Movement, Human and Health Sciences, University of Rome “Foro Italico”, Rome, Italy; 4 Department of Mechanical Engineering, The University of Sheffield, Sheffield, United Kingdom; 5 INSIGNEO Institute for in Silico Medicine, The University of Sheffield, Sheffield, United Kingdom; Universita degli Studi Magna Graecia di Catanzaro Scuola di Medicina e Chirurgia, ITALY

## Abstract

Interleukin-6 (IL-6) has been recently shown to play a central role in glucose homeostasis, since it stimulates the production and secretion of Glucagon-like Peptide-1 (GLP-1) from intestinal L-cells and pancreas, leading to an enhanced insulin response. In resting conditions, IL-6 is mainly produced by the adipose tissue whereas, during exercise, skeletal muscle contractions stimulate a marked IL-6 secretion as well. Available mathematical models describing the effects of exercise on glucose homeostasis, however, do not account for this IL-6 contribution. This study aimed at developing and validating a system model of exercise’s effects on plasma IL-6 dynamics in healthy humans, combining the contributions of both adipose tissue and skeletal muscle. A two-compartment description was adopted to model plasma IL-6 changes in response to oxygen uptake’s variation during an exercise bout. The free parameters of the model were estimated by means of a cross-validation procedure performed on four different datasets. A low coefficient of variation (<10%) was found for each parameter and the physiologically meaningful parameters were all consistent with literature data. Moreover, plasma IL-6 dynamics during exercise and post-exercise were consistent with literature data from exercise protocols differing in intensity, duration and modality. The model successfully emulated the physiological effects of exercise on plasma IL-6 levels and provided a reliable description of the role of skeletal muscle and adipose tissue on the dynamics of plasma IL-6. The system model here proposed is suitable to simulate IL-6 response to different exercise modalities. Its future integration with existing models of GLP-1-induced insulin secretion might provide a more reliable description of exercise’s effects on glucose homeostasis and hence support the definition of more tailored interventions for the treatment of type 2 diabetes.

## Introduction

The cytokine Interleukin-6 (IL-6) has a central role in glucose homeostasis; however, the evidence of its dual nature of as an adipokine (i.e., adipose tissue-derived cytokine) and as a myokine (i.e., muscle-derived cytokine) has been a matter of scientific debate [1]. Experimental and clinical evidences suggest that an altered secretion of adipokines by the adipose tissue determines a condition of “chronic low-grade inflammation”, correlated to insulin resistance and IL-6 as one of the adipokines involved in this process [2–5]. It has also been shown that IL-6 is secreted by skeletal muscles during exercise [6], in an amount that has proven to be considerable and depending on the intensity and duration of the exercise [7–9]. IL-6 has been shown to trigger the secretion of anti-inflammatory cytokines [10,11] and to contribute to an improvement of insulin-stimulated glucose disposal [12].

The dual nature of IL-6 has been unveiled by the recent observation that, as a response to changes in insulin demand, it mediates the crosstalk between insulin-sensitive tissues (muscle ad adipose tissue), intestinal L-cells, and pancreatic islets [13]. IL-6 release, either from the contraction of skeletal muscle or from white adipose tissue, stimulates the production and secretion of Glucagon-like Peptide-1 (GLP-1) from intestinal L-cells and pancreas. This leads to an enhanced insulin response and, thus, to improved glycemic levels [14]. As a consequence, increased levels of circulating IL-6 observed in chronic low-grade inflammation, may represent a compensatory mechanism to maintain glucose homeostasis in insulin resistant conditions [15].

Only a few mathematical models describing the effects of an exercise bout on hormones and metabolites involved in glucose homeostasis have been developed to date [16–22] but none of them accounts for IL-6 contribution. This might indeed limit the scope of applicability of these models. Thus, the aim of this study was to design a system model of the effects of physical exercise on plasma IL-6 dynamics, able to account for both the adipose tissue and the skeletal muscle release.

## Materials and methods

### Model formulation

**Fig 1:**
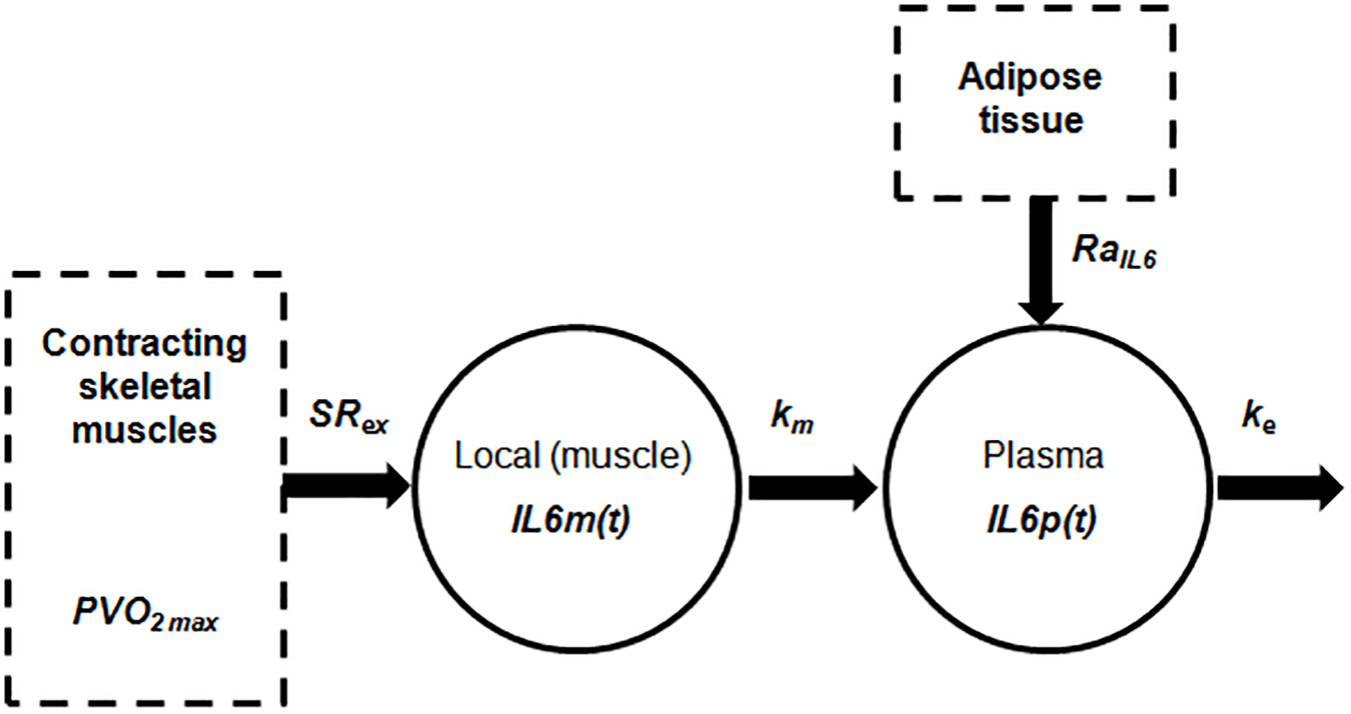
Two-compartment description of the IL-6 dynamics during exercise. Skeletal muscle secretes IL-6 in the local (muscle) blood flow (*IL6*_*m*_*(t)*) in response to change in oxygen consumption (*PVO*_*2max*_) with a secretion rate equal to *SR*_*e*x_. Plasma IL-6 (*IL6*_*p*_*(t)*) is the result of adipose tissue secretion (*Ra*_*IL6*_), hepatosplanchnic viscera removal (*k*_*e*_) and contribution coming from muscle compartment (through *k*_*m*_).

When at rest, subcutaneous and visceral adipose tissues are the principal responsibles for IL-6 production [23,24]. During physical exercise, on the contrary, IL-6 production is sustained by the skeletal muscle through intracellular signaling pathways. Depending on the duration and intensity of exercise [7], plasmatic IL-6 concentration increases exponentially [8] and then, as a result of this increase, the hepatosplanchnic viscera remove IL-6 from plasmatic circulation [25]. Consistently, it was postulated here that the IL-6 dynamics can be described by the model reported in Fig 1. Oxygen consumption provides a measure of exercise intensity and is usually quantified as percentage of the maximal oxygen uptake (VO_2max_, [26]). In this study it was hypothesized that oxygen consumption increases at the onset of exercise, reaches its target value within 5–6 minutes and then remains constant during the exercise; at the end of exercise, it is assumed to return to its basal value within 5–6 minutes, following the first order dynamics described in Roy [18].

The overall model is described by the following ordinary differential equations:

$$\frac{d{PVO}_{2max}(t)}{dt}=-0.8\cdot {PVO}_{2max}(t)+0.8\cdot u(t)\;{PVO}_{2max}(0)=0$$ (1)

$$\frac{d{IL6}_{m}(t)}{dt}={SR}_{ex}∙{PVO}_{2max}(t)-k_{m}∙{IL6}_{m}(t)\;{IL6}_{m}(0)=0$$ (2)

$$\frac{d{IL6}_{p}(t)}{dt}=k_{m}\cdot {IL6}_{m}(t)-k_{e}\cdot {IL6}_{p}(t)+\frac{{Ra}_{IL6}}{V}\;{IL6}_{p}(0)={IL6}_{b}$$ (3)

Eq (1) is taken from Roy et al. [18] and describes the dynamics of oxygen consumption for the whole exercise duration and the recovery as a linear first-order differential equation. *PVO*_*2max*_*(t)* represents the suprabasal oxygen consumption, expressed as percentage of VO_2max_. *u(t)* is the model input, representing the target value of the exercise intensity above the basal level:

$$u(t)=\left\{ \begin{array}{cc} 0 & 0<t<t_{ex}^{start}\\ T_{v} & t_{ex}^{start}\leq t\leq t_{ex}^{end}\\ 0 & t>t_{ex}^{end} \end{array}\right.$$ (4)

The target value *T*_*v*_ ranges from 0 to 92%, considering a basal consumption equal to 8% of the maximum value [27]. The coefficient 0.8 min^-1^ is chosen to allow *PVO*_*2max*_*(t)* to reach the target value *T*_*v*_ in 5–6 minutes. In Eq (2), *IL6*_*m*_*(t)* represents the IL-6 concentration in the muscle compartment. The first term on the right-hand side of Eq (2) accounts for muscle IL-6 increase from stationary conditions in response to muscle contraction during exercise, which is described as linearly dependent on *PVO*_*2max*_*(t)* through *SR*_*ex*_, representing the muscular IL-6 secretion rate. In Eq (3), *IL6*_*p*_*(t)* represents the IL-6 concentration in the plasma compartment. The first term of the right-hand side of Eq (3) accounts for the increase in the plasma IL-6 from its basal value (*IL6*_*b*_) due to IL-6 released from the skeletal muscle. The second term of Eq (3) represents the IL-6 removal from circulation after exercise operated by the hepatosplanchnic viscera. Lastly, the third term accounts for the IL-6 production rate during non-perturbed conditions, which is represented by the adipose tissue contribution (*Ra*_*IL6*_) normalized to the distribution volume *V*. For given values of *k*_*e*_, *V* and *IL6*_*b*_, the value of *Ra*_*IL6*_ was determined by imposing the steady-state condition (*dIL6*_*p*_*(t)/dt* = 0) representing the equilibrium of the dynamic system described by Eqs (1–3):

$${Ra}_{IL6}=k_{e}∙{IL6}_{b}∙V$$ (5)

The initial value of *IL6*_*b*_ in Eq (3) was set to the fasting pre-exercise value. The parameter *V* was assumed as fixed and assigned a numerical value taken from the literature [28]. The free model parameters *SR*_*ex*_, *k*_*m*_ and *k*_*e*_ were identified by fitting the IL-6 plasma concentration data during and after an exercise session as described in Parameter estimation.

### Datasets

Four experimental studies and relevant datasets were used to build and validate the model, differing from each other in terms of implemented exercise protocol and hence allowing to investigate the ability of the model to predict changes in IL-6 response due to changes in exercise intensity and duration.

- Dataset 1 (D_1_): Ostrowski et al. [29]. Venous blood was sampled in ten endurance-trained male athletes before, during and after (6 h recovery period) 2.5 h of treadmill running at 75% of their VO_2max_;
- Dataset 2 (D_2_): Fischer et al. [30]. Seven young healthy physically active non-athletes men performed three hours of a dynamic two-legged knee-extensor exercise at 50% of their individual maximal power output. Systemic arterial plasma IL-6 concentration was measured at 0, 30, 60, 120, 180, 210, 240, 300, 360 and 1380 minutes after the beginning of exercise;
- Dataset 3 (D_3_): Steensberg et al. [8]. Six young healthy not regularly training males performed five hours of a dynamic one-legged knee-extensor exercise at 40% of their individual maximal power output, and ~ 20% of their VO_2max_. Systemic arterial plasma IL-6 concentration was measured every hour during the exercise session;
- Dataset 4 (D_4_): Febbraio et al. [25]. Six healthy male subjects performed 120 min of semi-recumbent cycling at 62% of their VO_2max_. Systemic arterial plasma IL-6 concentration was measured immediately before exercise and at 30-min intervals during exercise.

In D_1_, D_3_ and D_4_ the target value for the exercise intensity was already expressed in terms of %VO_2max_, thus allowing to exactly set the model input *T*_*v*_. Conversely, an approximation was required for D_2_ to convert exercise intensity from percentage of maximal power output to %VO_2max_. The target value for exercise intensity *T*_*v*_ was set considering the conversion reported in [31]. The values of *T*_*v*_, *IL6*_*b*_ and exercise duration for simulating the exercise protocols are summarized in Table 1.

**Table 1:** Values of *IL6*_*b*_, *T*_*v*_ and exercise duration.

Study	IL6_b_	T_v_	Exercise duration
	pg·ml^-1^	%	min
D_1_: Ostrowski et al. [29]	1.7	67	150
D_2_: Fischer et al. [30]	1.7	47	180
D_3_: Steensberg et al. [8]	0.9	12	300
D_4_: Febbraio et al. [25]	1.8	54	120

### Parameter estimation

The estimation of the model parameters *SR*_*ex*_, *k*_*m*_ and *k*_*e*_ was performed by fitting literature mean values of plasma IL-6 concentration, using a weighted non-linear least squares approach. The weighted residual sum minimization procedure was performed by using the Levenberg–Marquardt algorithm implemented in the *lsqnonlin* Matlab^®^ (The MathWorks, Natick, MA, USA) function. Each element of the diagonal of the weight matrix was set to be equal to the reciprocal of the error variance of the IL-6 measurement. The errors in IL-6 measurements were assumed to be normally distributed random variables, with zero mean and a constant percent coefficient of variation (assumed equal to 6.9%, obtained by averaging the IL-6 intra-assay coefficients of variation reported in the considered datasets). The precision of the estimate of each parameter was expressed using the percent coefficient of variation, CV% = (SD_pi_ ∕ p_i_), where the standard deviation SD_pi_ was derived from the inverse of the Fisher information matrix and pi is the corresponding parameter estimate [32].

### Cross-validation for parameters selection

The ideal set of parameters would be the one maximizing the ability of the model to reproduce a dataset that is independent of the data that have been used to originally train it. A K-fold cross-validation procedure was used to determine which, among the K considered datasets, provides the “best” set of estimated parameters according to this criterion.

The K-fold cross-validation procedure included four stages, one per dataset D_k_ (with k = 1,…,4). At each stage of the procedure, the investigated D_k_ was used as the training dataset to estimate *SR*_*ex*_, *k*_*m*_ and *k*_*e*_ (as described in Parameter estimation), while the remaining three datasets were used for the validation. The model predictions used for the validation were calculated using these estimated parameters, together with the *IL6*_*b*_ value and model input (duration and intensity of the exercise) from the corresponding protocol (Table 1). The total number of observations available was $N=\sum_{k=1}^{K}n_{k}$ where n_k_ is the number of observations in each D_k_.

At each stage, the Mean Squared Prediction Error (MSPE) for each dataset was computed as:

$${MSPE}_{k}=\frac{1}{n_{k}}\sum_{j=1}^{n_{k}}\left(y_{j}-y_{j}^{*}\right)$$ (6)

where y_j_ is j-th experimental observation and y_j_^*^ is the j-th model prediction. The Cross-Validation Prediction Error Estimate (CVPEE) for each stage of the procedure was then computed as:

$$CVPEE=\frac{1}{N}\sum_{k=1}^{K}n_{k}∙{MSPE}_{k}.$$ (7)

The “best set” of estimated parameters was chosen as the one providing the lowest CVPEE.

## Results

Results of the cross-validation are reported in Table 2. In all four stages, the value of *V* (IL-6 distribution volume) was set to 14 l according to reported experimental data [28]. The half-life duration corresponding to the turnover rate *k*_*e*_ was 13 min for D_1_, 56 min for D_2_, 147 min for D_3_ and 51 min for D_4_.

**Table 2:** Results of the cross-validation procedure.

Stage	Estimation set	Estimated parameters			Sum of CV%	CVPEE	
		SR_ex_ (CV%)	k_m_ (CV%)	k_e_ (CV%)			Ra_IL6_
		pg·ml^-1^·min^-1^	min^-1^	min^-1^			pg·min^-1^
1	D_1_	0.045 (7)	0.004 (10)	0.053 (<1)	<18	17.5	1,261
2	D_2_	0.014 (9)	0.002 (<1)	0.012 (11)	<21	48.6	296
3	D_3_	0.038 (171)	0.0002 (189)	0.0047 (92)	452	107	58
4	D_4_	0.637 (<1)	0.00008 (23)	0.013 (74)	<98	86.2	340

**Fig 2:**
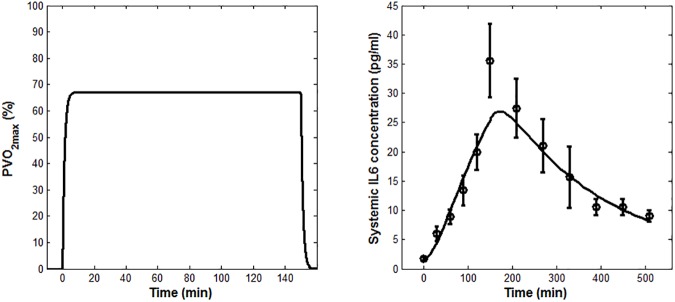
Model fit results. (A) *PVO*_*2max*_ model prediction (B) Mean model fit (solid line) for IL-6. Measured IL-6 concentrations (means ± SEM) from Ostrowski et al. [29] are shown, along with the model fit.

**Fig 3:**
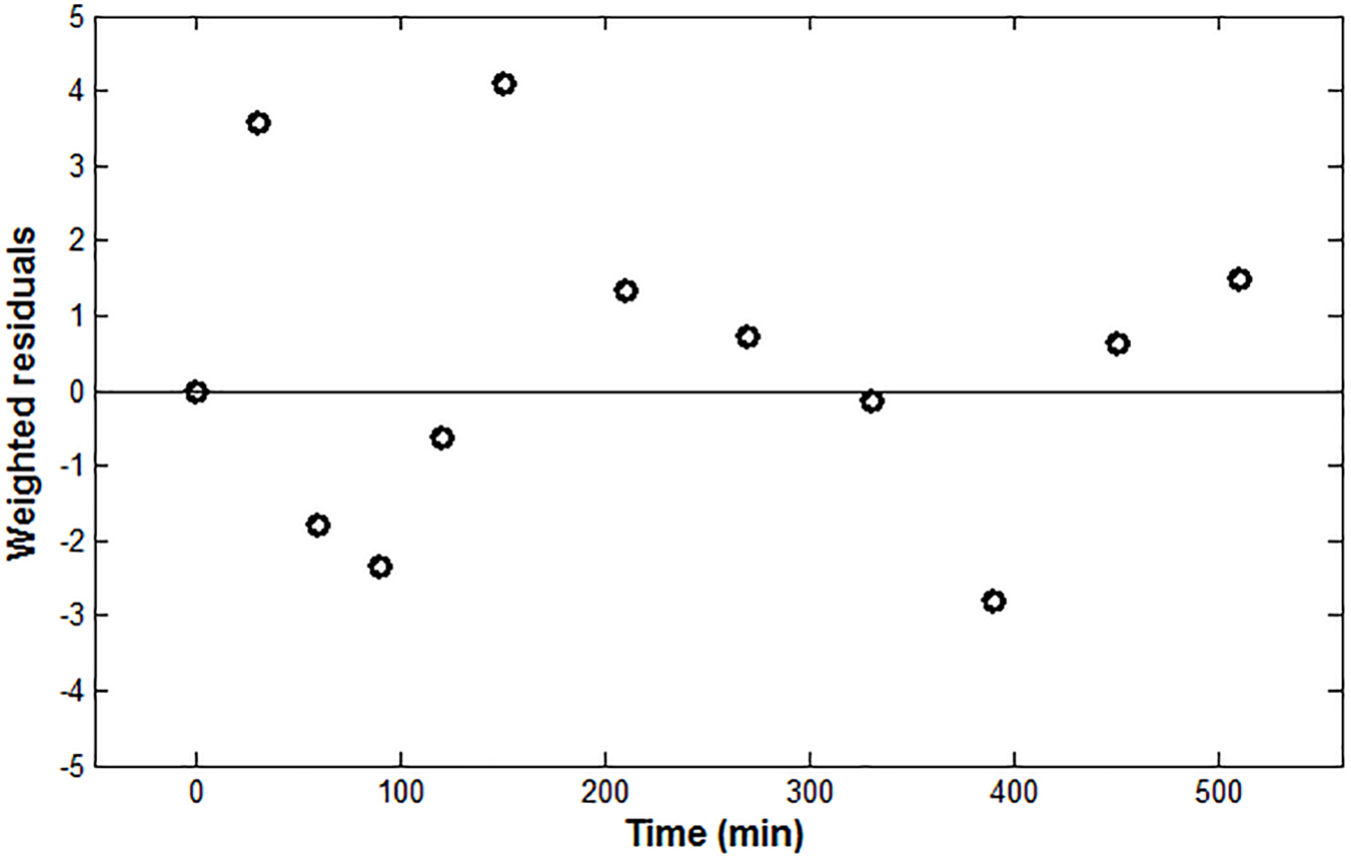
IL-6 weighted residuals.

According to the results reported in Table 2, the lowest CVPEE was obtained at Stage 1, using dataset D_1_ as estimation set. Stage 1 was also characterized by having the lowest sum of CV% associated to the estimations of *SR*_*ex*_, *k*_*m*_ and *k*_*e*_. Accordingly, the data from Ostrowski et al. [29] were selected as the one to be used for the parameter estimation. The mean of experimental IL-6, together with the IL-6 model prediction as fitted applying Eqs (1–3), are shown in Fig 2. The time course of the weighted residuals is shown in Fig 3.

**Fig 4:**
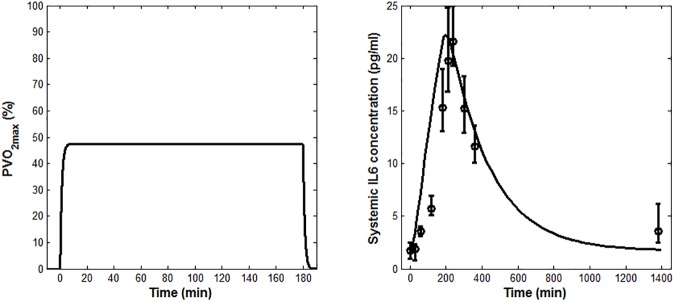
Model validation results obtained using the conditions reported in the study by Fischer et al. (A) *PVO*_*2max*_ model prediction (B) Measured IL-6 concentrations (means ± SEM) from Fischer et al. [30], shown along with the model prediction (solid line).

**Fig 5:**
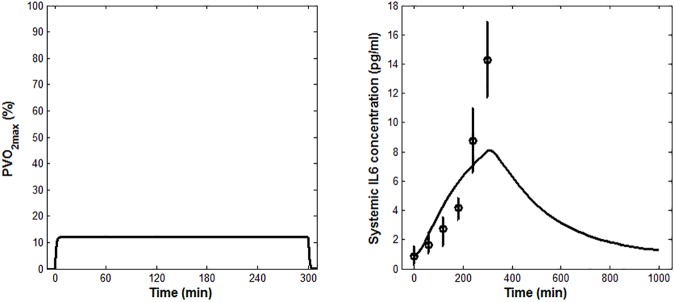
Model validation results obtained using the conditions reported in the study by Steensberg et al. (A) *PVO*_*2max*_ model prediction (B) Measured IL-6 concentrations (medians and quartiles) from Steensberg et al. [8], shown together with the model prediction (solid line).

**Fig 6:**
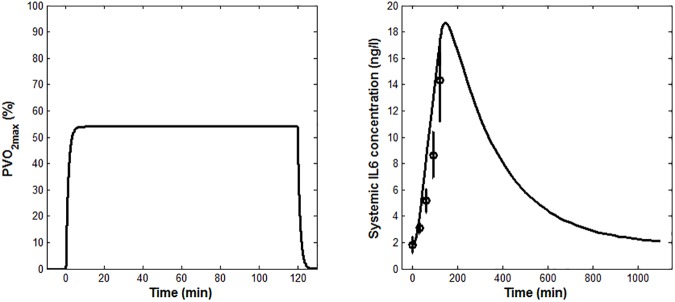
Model validation results obtained using the conditions reported in the study by Febbraio et al. (A) *PVO*_*2max*_ model prediction (B) Measured IL-6 concentrations (means ± SEM) from Febbraio et al. [25], shown together with the model prediction (solid line).

After the fitting with D_1_, the model was validated against the other three datasets. Figs 4A, 5A and 6A show the model predictions of the oxygen consumption kinetics. Figs 4B, 5B and 6B show the IL-6 concentrations obtained from the model validation, plotted alongside the experimental data. The simulations of the IL-6 dynamics, ran imposing the initial conditions used in [30], [8], [25] (*IL6*_*b*_ values summarized in Table 1), led to computed values for *Ra*_*IL6*_ of 1,252 pg/min, 809 pg/min and 1,335 pg/min, respectively.

## Discussion

This study aimed at proposing and validating a system model describing the IL-6 dynamics during an exercise bout. A preliminary single-compartment description has been proposed in [33], however the novel two-compartment formulation here reported provides a more detailed and physiologically sound description of the IL-6 dynamics within the skeletal muscle.

Although it’s typically assumed that both the exercise modality and the associated number of engaged muscles play a role in determining IL-6 response [8,34,35], recent studies showed that different exercise modalities with the same relative intensity (i.e. same %VO_2max_), may result in similar absolute IL-6 releases [36]. For this reason, none of the model parameters were designed to account for differences in the amount of muscles engaged in the exercise. As a result, the model requires as an input only one target value of maximum oxygen consumption, i.e., one value of %VO_2max_. This notably simplifies the mathematical description of the exercise and, without a significant loss of precision, allows flexibility in predicting the IL-6 response to different kind of exercise (running, cycling, etc.). This is a notable advantage with respect to the single-compartment formulation [33], which requires a continuous measurement of the heart rate.

The model-fit results, reported in Fig 2B, showed that the model is able to capture well the experimental plasma IL-6 time course, although its peak value was slightly underestimated. This is probably due to the chosen definition of the dynamics of the oxygen consumption, which was simplistically represented as a first-order differential equation (Eq (1)), even though the data used for the parameter estimation come from athletes performing a heavy-intensity exercise. As suggested in the literature [37], in fact, at least two kinetic components should be used to characterize the oxygen uptake response dynamics to a heavy-intensity exercise. Nonetheless, this choice did not seem to affect the model credibility, as inferred from the fact that the weighted residuals reported in Fig 3 were substantially randomly distributed.

The selected set of parameters represents the “best set” according to the cross-validation procedure and provided plausible estimates, as confirmed by comparison with previously published data. In fact, the IL-6 half-life has been previously found to range between 5 to 11 min [28,38] and a turnover rate *k*_*e*_ of 0.053 min^-1^, corresponding to a half-life of 13 min when considering a distribution volume of 14 l, was here estimated. Subcutaneous abdominal adipose tissue IL-6 release in resting conditions has been previously reported to be 3.84 pg/100 g adipose tissue/min [24]. Assuming that subcutaneous and visceral adipose tissue masses release IL-6 at the same rate, for a non-obese individual, an IL-6 production rate for the whole body adipose tissue mass (subcutaneous and visceral) of about 1 ng/min can be extrapolated [24]. This latter value is consistent with the range of *Ra*_*IL6*_ (0.8 ÷1.3 ng/min) values obtained in the present study. The *Ra*_*IL6*_ values remained physiologically meaningful (ranging from 1,080 to 1,450 pg/min) even when accounting for *IL6*_*b*_ variability (expressed as mean ± SEM) in the dataset selected for parameter estimation. The estimates of *Ra*_*IL6*_ and *k*_*e*_ provided by the other three datasets, conversely, were not physiologically meaningful.

The IL-6 secretion rate from adipose tissue increases at the end of a moderate exercise bout and then remains higher for a prolonged post-exercise period [23]. The fact that this value was set as constant within the model here investigated, should not have affected its accuracy, since the relative contribution to systemic IL-6 level from organs other than the contracting skeletal muscles is negligible during and after exercise [28]. Further studies are needed to fully corroborate this hypothesis.

The model reproduced well the effects on plasma IL-6 concentration due to changes in the exercise duration and intensity. Its predictions were less accurate for low- than for high-intensity exercise protocols (Fig 5 vs. Figs 4 and 6), for which the predicted peak value fell outside the range of experimental values. The model prediction, however, might still be considered as acceptable in light of the fact that previous studies on IL-6 response to low-intensity exercise reported no increase during exercise [10,39]. Notably, the deviation of the model output from the experimental data at the beginning of the exercise (Fig 4 and Fig 6) does not affect the overall IL-6 dynamics and the extent of the IL-6 increase. Such deviation could be ascribed to differences among the subjects considered in the three chosen validation studies and, more specifically, to the effect that the training status and the relevant muscular glycogen content might have had on the IL-6 dynamics [34,35].

GLP-1-based clinical therapies have firmly established their importance among the therapeutical approaches available for the treatment of type 2 diabetes [40–42]; their action is exerted by potentiating glucose-stimulated insulin secretion. Interestingly, GLP-1 secretion increases in an IL-6-dependent manner [14], but also as a function of glucose transit into the gastrointestinal tract [43]. Whereas mechanistic models enabling the quantitation of insulin response to GLP-1, as well as models of GLP-1-mediated insulin response to glucose transit into the gastrointestinal tract [44–49] have been previously proposed, the GLP-1 response to IL-6 has never been modeled. The system model here proposed could be seen as an important step toward the modeling of this response, and as such as a step toward the improvement of GLP-1-based clinical therapy. An integrated description of the GLP-1-induced insulin secretion, accounting for the contribution of exercise-induced IL-6 and of the meal-induced glucose transit into the gastrointestinal tract could allow to reliably describe the response to exercise in real-life situations. More importantly, this approach could be used to quantify the combined effects of exercise and meal on glucose homeostasis regulation, hence favoring the definition of a tailored exercise-based intervention in the control of insulin secretion for the treatment of type 2 diabetes. Future research is recommended in this direction.

In conclusion, this study proposes an innovative two-compartment system model of the effects of an exercise bout on IL-6 dynamics. The model is able to describe data from various exercises, it is precise in the parameter estimation, and it is in good agreement with published values of various experimentally measured physiological quantities.
